# Macrolide- and Telithromycin-resistant *Streptococcus pyogenes*, Belgium, 1999–2003^1^

**DOI:** 10.3201/eid1106.041247

**Published:** 2005-06

**Authors:** Surbhi Malhotra-Kumar, Christine Lammens, Sabine Chapelle, Monique Wijdooghe, Jasper Piessens, Koen Van Herck, Herman Goossens

**Affiliations:** * University of Antwerp, Antwerp, Belgium

**Keywords:** molecular epidemiology, antibiotic resistance, bacterial, pediatric, pharyngitis, clones

## Abstract

We found a 13% macrolide resistance in 3,866 *Streptococcus pyogenes* isolated from tonsillopharyngitis patients; 59% macrolide-resistant isolates were distributed in 5 clones, suggesting the importance of both resistance gene transfer and clonal dissemination in the spread of these organisms. We also report one of the largest collections of telithromycin-resistant isolates.

*Streptococcus pyogenes* causes several million cases of upper respiratory tract infection each year. The problem of these infections is growing as resistance increases among *S. pyogenes* to the macrolide group of antimicrobial drugs commonly used to treat such infections (1–4). *S. pyogenes* acquires resistance by 2 main mechanisms. The first is active drug efflux mediated by an ATP-binding cassette transporter wherein *mef*(A) encodes the transmembrane domains and *msr*(D) encodes the ATP-binding domains (5). This pattern of resistance is demonstrated by an M phenotype. In the second mechanism, gene products of *erm*(B) or *erm*(A) methylate the macrolide-binding site on 23S rRNA and stall bacterial protein synthesis. This pattern of resistance is demonstrated by either a constitutive (cMLS) or an inducible (iMLS) phenotype. A third, rare, mechanism is modification of the drug binding site on rRNA by mutation that is expressed as an M or a cMLS phenotype. The newest generation of macrolides, the ketolides, are also active against macrolide-resistant strains; however, few *S. pyogenes* strains of the cMLS phenotype have been found to be ketolide resistant (6).

In Belgium, the first ketolide to be used clinically, telithromycin, was approved in October 2002 to treat community-acquired respiratory infections in patients >12 years of age. We investigated the temporal trends in resistance and clonality among macrolide (including telithromycin)-resistant *S. pyogenes* recovered from patients with tonsillopharyngitis during surveillance studies conducted in Belgium.

## The Study

During 1999–2003, a total of 4,031 nonduplicate, putative *S. pyogenes* isolates were collected from 10 Belgian provinces at the reference center with the date of isolation, specimen source, and patient's age and residential address. By using a battery of tests, for example, β-hemolysis on blood agar, Gram stain, catalase production, pyrrolidonyl arylamidase, presence of Group A antigen, and bacitracin susceptibility, 3,866 isolates were confirmed to be *S. pyogenes*. The age of the patient was known in 3,654 cases. Population statistics are detailed in the first half of Table 1.

**Table 1:** Yearly prevalence of Streptococcus pyogenes isolates screened and of macrolide-resistant S. pyogenes distributed by patient age group and isolate phenotype, 1999—2003

	1999	2000	2001	2002	2003
Total *S. pyogenes* screened	598	336	633	1,226	1,073
Isolated from adults (mean age, 34.7 y; SD, 11.1 y; range, 17 to 91 y)	220 (36.7%)	144 (43.1%)	245 (38.7%)	469 (38.2%)	453 (42.0%)
Isolated from children (mean age, 7.2 y; SD, 3.5 y; range, 3 mo to 16.9 y)	357 (59.6%)	172 (51.2%)	367 (58.0%)	675 (55.0%)	552 (51.0%)
Macrolide-resistant *S. pyogenes*	81 (14%)	41 (12%)	73 (12%)	215* (18%)	96* (9%)
Isolated from adults	23 (4%)	16 (5%)	29 (5%)	82 (7%)	38† (4%)
Isolated from children	56 (9%)	22 (7%)	44 (7%)	126‡ (10%)	50† (5%)
Constitutive phenotype	49/81 (8%)	10/41§ (3%)	28/73 (4%)	68/215 (6%)	54/96 (5%)
M phenotype	32/81 (5%)	29/41 (9%)	39/73 (6%)	141/215 (12%)	38/96§ (4%)
Inducible phenotype	–	2/41 (1%)	6/73 (1%)	7/215 (1%)	4/96 (0.4%)

*Increase and decrease in macrolide resistance from 2001 to 2002 and from 2002 to 2003, respectively, was significant (p<0.001). †Prevalence of macrolide-resistant *S. pyogenes* decreased significantly among both children and adults from 2002 to 2003 (p<0.0001). ‡Prevalence of macrolide-resistant *S. pyogenes* increased significantly among children from 2001 to 2002 (p = 0.005). §Decrease in prevalence of cMLS isolates from 1999 to 2000 (p = 0.005) and of M phenotype isolates from 2002 to 2003 (p<0.0001) was significant. Pearson's χ^2^-test with Bonferonni post-hoc adjustments was used for all multiple comparisons. p<0.05 (2-sided) was significant.

By using erythromycin (78 μg) and clindamycin (25 μg) double-disk diffusion (Neo-Sensitab discs; Rosco, Taastrup, Denmark), all 3,866 *S. pyogenes* isolates were further screened for a phenotypic expression of macrolide resistance, which was identified in 506 (13%) isolates. The proportion of macrolide-resistant isolates among the total *S. pyogenes* isolated from each of the 10 Belgian provinces fluctuated from 0% to 40% over the 5 years studied. The 3 known phenotypes, cMLS, iMLS, and M, were identified in 209 (41%), 18 (4%), and 279 (55%) isolates, respectively. Changes in prevalence of the 3 phenotypes among macrolide-resistant *S. pyogenes* over 5 years are presented in the second half of Table 1.

MICs of erythromycin, clindamycin, tetracycline (Sigma-Aldrich, St. Louis, MO, USA), clarithromycin (Abbott, Louvain-la-Neuve, Belgium), azithromycin (Pfizer, Groton, CT, USA), and telithromycin (Aventis, Romainville, France) were further determined by agar dilution (7). Susceptible and resistance breakpoints for telithromycin were ≤1 μg/mL and ≥4 μg/mL, respectively. Briefly, a 10^4^ CFU/spot inoculum was incubated at 37°C for 18–24 h in ambient air. The MIC profiles of the 3 macrolide-resistant phenotypes to various antimicrobial drugs are presented in the Table A1. The yearly prevalence (1999–2003) of telithromycin resistance (MIC ≥4 μg/mL) among macrolide-resistant *S. pyogenes* was 2%, 7%, 11%, 13%, and 10%, respectively. Thus, the total telithromycin-resistant isolates (N = 50) identified here constitute the largest collection reported. Of the 50 telithromycin-resistant *S. pyogenes*, 49 belonged to the cMLS and 1 to the iMLS phenotype. These isolates exhibited erythromycin MICs of 128–>512 μg/mL. Thirty (60%) telithromycin-resistant *S. pyogenes* were isolated from children, of which 28 (56%) were ≤12 years of age.

We further investigated clonality in all macrolide-resistant isolates and in a random selection of 331 macrolide-susceptible isolates by pulsed-field gel electrophoresis (PFGE) and *emm* typing on reverse line blotting as described previously (1). PFGE was performed by using *Sma*I; however, for most *me*f(A)-positive isolates that proved refractory to *SmaI* restriction, *SfiI* restriction was utilized. PFGE patterns were analyzed by using GelCompar software 4.0 (Applied Maths, Kortrijk, Belgium). The 506 macrolide-resistant *S. pyogenes* were typed into 17 *emm* types and 76 PFGE types, of which 53 (70%) types were distributed among M phenotype isolates (see Table A2). Ratios of PFGE types to number of *S. pyogenes* isolates were 0.18 and 0.09 for the M and cMLS phenotypes, respectively. Table 2 shows the temporal evolution over 5 years of the 5 major cMLS and M phenotype clones. Clones 1, 4, and 23 constituted 99%, 98%, and 100% of all the macrolide-resistant *emm*22, *emm*28, and *emm*11, respectively, isolated during the course of this study, while clones 1,001 and 1,002 constituted 97% and 39% of the *emm*1 and *emm*4 macrolide-resistant *S. pyogenes* serotypes, respectively. Serotypes *emm*1, *emm*4, *emm*11, *emm*22 and *emm*28 formed 70% of the total macrolide-resistant *S. pyogenes*. Among the 331 macrolide-susceptible *S. pyogenes* analyzed, the prevalence of clones 1, 4, 23, 1,001, and 1,002 was 5% (n = 18), 1% (n = 3), 0.3% (n = 1), 2% (n = 5), and 0.3% (n = 1), respectively (data not shown). Telithromycin resistance was distributed among 7 cMLS serotypes (*emm*22, *emm*28, *emm*11, *emm*12, *emm*77, *emm*6, and, *emm*2).

**Table 2:** Temporal changes in the distribution of major pulsed-field gel electrophoresis and *emm* types among the 3 macrolide-resistant *Streptococcus pyogenes* phenotypes*

Macrolide-resistant phenotype	Pulsed-field gel electrophoresis cluster (*emm* type)	Frequency (n = 506)	No. (%) of macrolide-resistant *S. pyogenes*				
			1999 (n = 81)	2000 (n = 41)	2001 (n = 73)	2002 (n = 215)	2003 (n = 96)
Constitutive	1 (*emm*22)	70	45 (56%)	7 (17%)†	9 (12%)	7 (3%)	2 (2%)
	4 (*emm*28)	45	–	–	4 (5%)	15 (7%)	26 (27%)
	23 (*emm*11)	28	–	–	1 (1%)	6 (3%)	21 (22%)
M	1001 (*emm*1)	128	7 (9%)	12 (29%)	23 (32%)	80 (37%)‡	6 (6%)‡
	1002 (*emm*4 )	28	2 (2.5%)	2 (5%)	7 (10%)	7 (3%)	10 (10%)

*A ≤6-band difference was employed to assign isolates to a clone according to Tenover et al. (8). PFGE clusters up to 100 designate restriction with *Sma*I and clusters ≥1,000 designate restriction with *Sfi*I. †Decrease in prevalence of the 1/*emm*22 clone from 1999 to 2000 was highly significant (p<0.001). ‡Both the increase and decrease in prevalence of the 1001/*emm*1 clone from 2001 to 2002 and from 2002 to 2003, respectively, were significant (p<0.01).

We next studied the genotype for the 3 macrolide-resistant phenotypes. Polymerase chain reaction was performed for *erm*(A), *erm*(B), and *mef*(A) (1,9,10). Isolates negative for all 3 genes were screened for ribosomal mutations in L4, L22, and portions of 23S RNA genes by using published primers (11). Amplimers were analyzed by direct double-strand sequencing (3730 DNA Analyzer, Applied Biosystems, Foster City, CA, USA) with the BigDye Terminator Version 3.1 Kit (Applied Biosystems). Nucleotide sequence alignment was done with SeqMan (DNASTAR Inc., Madison, WI, USA). Phenotypic and genotypic profiles of the macrolide-resistant *S. pyogenes* were generally consistent; however, 3% of the resistant isolates carried 2 resistance genes. Of the 209 cMLS isolates, 199 carried *erm*(B), 9 carried *erm*(B)+*mef*(A), and 1 carried *erm*(B)+*erm*(A). Of the 279 M phenotype isolates, 273 carried *mef*(A), 1 carried *erm*(B)+*mef*(A), and 4 carried *mef*(A)+*erm*(A). The 1 isolate that was negative for all 3 genes carried a single A2059G (*Escherichia coli* numbering system) mutation in the 23S rRNA gene. The A2059G mutation, although quite frequent in *S. pneumoniae*, has been rarely observed in *S. pyogenes*. Finally, of the 18 iMLS isolates, 5 carried *erm*(B) and 13 carried *erm*(A).

Ten percent of the macrolide-resistant strains harboring *erm*(B) alone or with *mef*(A) were also telithromycin-resistant, and telithromycin has additional binding sites on 23S rRNA. Therefore, we hypothesized that either mutations in the *erm* gene promoter region have upregulated methylase expression or that mutations in the coding region have changed the methylase specificity to include the additional binding sites of telithromycin. Alternatively, mutations at the additional binding sites on the 23S rRNA genes might also disable the binding arm; however, a recent study described only a low level of telithromycin resistance in the presence of these mutations (12). Utilizing 3 pairs of overlapping primers (primer sequences available on request), DNASTAR software, and sequence data of *Tn1545* (National Center for Biotechnology Information, Rockville, MD, USA, accession no. X52632), the entire *erm*(B) gene, including the promoter and control peptides, were sequenced from 10 telithromycin-resistant isolates. In addition, L4, L22, and portions of 23S rRNA genes were also amplified as above. Analysis of the sequencing data showed a single H118R (A677G) substitution in the *erm*(B) coding region of all 10 telithromycin-resistant isolates. While our study was ongoing, the H118R substitution in *erm*(B) was also confirmed independently for 2 telithromycin-resistant isolates (6).

## Conclusions

We demonstrated in this study that overall macrolide resistance in Belgium is driven by an epidemic spread of a few major clones as well as by resistance gene transfer among genetically diverse *S. pyogenes*. On average, we demonstrated a 2-fold (13%) increase in macrolide resistance in Belgium from 1999 to 2003, compared to that observed from 1995 to 1997 (6.5%) (1). Although, a general increase in macrolide-resistance in Europe has been observed during the last few years, resistance levels tend to differ considerably between countries. For instance, while resistance rates in Germany (6) and Poland (4) were similar to those observed in Belgium, considerably higher resistance levels were observed in Spain and Portugal (2), as well as in Italy (3). Provincial variations in macrolide-resistance observed in Belgium have also been reported in other countries (3); however, the precise causes underlying such variations within or between countries are not fully understood. Macrolide consumption might be one factor that explains the regional variations in macrolide-resistant *S. pyogenes* in Spain and Finland (13,14), especially when consumption surpasses a critical threshold (15). However, in France, one of the highest macrolide consumption within Europe is not paralleled by an equally high resistance in *S. pyogenes* (16,17). The identification of telithromycin-resistant *S. pyogenes* in our study, many of which were already present in the community before the introduction of telithromycin in Belgium, also suggest that antimicrobial drug use and development of resistance might be dissociated to some extent. Clearly, other factors like natural fluctuations in prevalence of clones (18), patient compliance with antimicrobial drug regimens, fitness costs of drug resistance, or even tetracycline consumption (tetracycline and macrolide-resistance genes cosegregate) (19) might be important determinants for the development and spread of macrolide-resistant *S. pyogenes*. Thus, any direct link between macrolide use and resistance in *S. pyogenes* should be interpreted cautiously.

## Appendix

**Table A1:** In vitro activity of 6 antimicrobial agents against macrolide-resistant Streptococcus pyogenes phenotypes during 1999–2003

Antimicrobial*	Constitutive (n = 209)			M (n = 279)			Inducible (n = 18)		
	Range	MIC_50_	MIC_90_	Range	MIC_50_	MIC_90_	Range	MIC_50_	MIC_90_
Erythromycin	4–>512	>512	>512	1–128	8	16	1–>512	4	>512
Clindamycin	2–>512	>512	>512	≤0.03–4	0.06	1	0.125–256	0.75	128
Clarithromycin	1–>512	>512	>512	1–256	8	16	0.5–>512	2	>512
Azithromycin	4–>512	>512	>512	1–128	8	16	0.5–>512	4	>512
Tetracycline	0.06–128	32	64	≤0.03–64	0.25	0.5	0.06–64	0.5	64
Telithromycin†	0.0075–32	1	8	0.0075–2	0.5	0.5	0.003–4	0.06	0.5

*Dilution range of 0.03 to 512 μg/mL for all antimicrobial agents except telithromycin, which is 0.00375 to 64 μg/mL. †Breakpoints for telithromycin, susceptible ≤1μg/mL, resistant ≥4 μg/mL.

**Table A2:** Temporal changes in pulsed-field gel electrophoresis (PFGE) and emm type distribution among 3 macrolide-resistant Streptococcus pyogenes phenotypes

Macrolide-resistant phenotype	*Emm* type	PFGE cluster*	Frequency (n=506)	No. (%) of macrolide-resistant *S. pyogenes*				
				1999 (n=81)	2000 (n=41)	2001 (n=73)	2002 (n=215)	2003 (n=96)
cMLS	*emm*22	1	70	45 (56)	7 (17)	9 (12)	7 (3)	2 (2)
		1057	1				1 (0.4)	
	*emm*4	10	9			9 (12)		
		11	8	3 (4)		2 (3)	2 (1)	1 (1)
		1002	1					1 (1)
		1009	2		2 (5)			
	*emm*76	32	1	1 (1)				
	*emm*2	new2	1		1 (2)			
	*emm*28	4	45			4 (5)	15 (7)	26 (27)
	*emm*11	23	28			1 (1)	6 (3)	21 (22)
	*emm*75	15	1			1 (1)		
	*emm*6	5	1			1 (1)		
	*emm*12	6	1			1 (1)		
		57	11				8 (4)	3 (3)
	*emm*77	10	6				6 (3)	
	NT†	2	3				3 (1)	
		49	12				12 (6)	
		64	4				4 (2)	
		65	1				1 (0.4)	
		69	2				2 (1)	
		70	1				1 (0.4)	
M	*emm*1	1001	128	7 (9)	12 (29)	23 (32)	80 (37)	6 (6)
		1022	2	2 (2)				
		3	1	1 (1)				
	*emm*12	6	1				1 (0.4)	
		1011	10	4 (5)	2 (5)	3 (4)	1 (0.4)	
		1025	10	3 (4)			2 (1)	5 (5)
		1026	2	2 (2)				
		1027	1	1 (1)				
		1030	2		1 (2)		1 (0.4)	
		1035	9			1 (1)	4 (2)	4 (4)
		1044	1				1 (0.4)	
		1047	2				2 (1)	
	*emm*2	18	1	1 (1)				
		1038	1	1 (1)				
		new 3	1	1(1)				
		1042	1				1 (0.4)	
		1034	1			1 (1)		
		1049	1				1 (0.4)	
	*emm*4	1002	28	2 (2.5)	2 (5)	7 (10)	7 (3)	10 (10)
		1007	2		2 (5)			
		1008	1	1 (1)				
		1009	1		1 (2)			
		1010	11	1 (1)	3 (7)	2 (3)	2 (1)	3 (3)
		1019	1	1 (1)				
		1021	2	2 (2)				
		1043	1				1 (0.4)	
		1044	3					3 (3)
		1054	2				2 (1)	
		1059	1				1 (0.4)	
	*emm*77	1028	4		4 (10)			
		9	2		1 (2)	1 (1)		
		10	3				3 (1)	
	*emm*75	1036	1			1 (1)		
		1060	1				1 (0.4)	
	*emm*33	1046	2				2 (1)	
		1052	2				2 (1)	
	*emm*78	1042	1				1 (0.4)	
	*emm*28	1033	1					1 (1)
	*emm*9	1042	2					2 (2)
	*emm*60	8	1	1 (1)				
	NT	42	1	1 (1)				
		1018	1		1 (2)			
		62	3				3 (1)	
		71	1				1 (0.4)	
		1005	2				2 (1)	
		1012	2				2 (1)	
		1033	2				2 (1)	
		1042	1				1 (0.4)	
		1043	3				3 (1)	
		1044	3				3 (1)	
		1045	1				1 (0.4)	
		1049	3				3 (1)	
		1050	1				1 (0.4)	
		1051	1				1 (0.4)	
		1055	1				1 (0.4)	
		1058	1				1 (0.4)	
		1061	2					2 (2)
		83	1					1 (1)
		26	1					1 (1)
iMLS	*emm*1	1031	1		1 (2)			
	*emm*22	1	2			2 (3)		
	*emm*4	10	1			1 (1)		
	*emm*9	61	1			1 (1)		
	*emm*77	9	2			1 (1)	1 (0.4)	
		10	1				1 (0.4)	
		50	2			1 (1)	1 (0.4)	
	*emm*12	57	1				1 (0.4)	
	*emm*18	1048	1				1 (0.4)	
	*emm*89	77	1					1 (1)
	NT	new 1	1		1 (2)			
		63	1				1 (0.4)	
		49	3					3 (3)

*PFGE profile numbers from 1-100 designate restriction with *SmaI* and above 1,000 restriction profiles generated with *SfiI.* †NT, non-typeable.
